# The Way of Attainment

**Published:** 1920-08-21

**Authors:** 


### THE WAY OF ATTAINMENT.

A matron, and member of the College of Nursing, sends us the following :—

It is one of Nature's favourite anomalies that the precursor of far-reaching reforms must ever be dissatisfaction and unrest; the spirit of restlessness must be set stirring beneath the weight of apathetic indifference before the bright flame of understanding can be kindled to illuminate mental darkness and order be evolved from chaos. If we apply this maxim to the present condition of the nursing services even the most pessimistic amongst us will experience a glimmer of hope.

There are professions, even as there are individuals, that find self-expression difficult, their training tends to reticence, their work to self-repression, they continue to hibernate in the channels indicated for them by their predecessors until changing conditions render their position untenable, and they realise that they must either assert themselves or cease to exist. This is the position to-day of the nursing services. From the early days of their inception they have lacked concerted expression. Nurses have complained bitterly amongst themselves, but they have never threshed out their grievances. They have been defective in the power of co-ordination, and they have failed to state their case clearly to the public because they have as yet evolved no definite policy.

Now the general public stands in the same relation to the nursing services as an employer to his employees, and no employer is prepared to improve economic conditions, to shorten working hours, and to grant facilities for higher education unless he is convinced that his employees desire and will appreciate these privileges—nay more, that they are determined to press their wishes by every available constitutional method. Distrust and suspicion are synonymous with misunderstanding, and unless members of the nursing services can agree amongst themselves as to their vital necessities and present a clear and concise statement for public consideration, they cannot hope to inspire the national mind with that confidence and sympathetic understanding which must be the forerunner of any alleviation of existing conditions.

The social and political economy of modern life is now so complex that no section of the community can claim complete independence of action, and public servants tend more and more to be controlled by communal thought; especially does this principle apply to the members of the nursing services. Nurses are drawn into the most intimate relations of life; they are present during the most poignant moments of human suffering and human joy; no scientific invention can replace them; they hold a unique position created for them by the dire need of humanity. The public are neither indifferent nor ungrateful; they are merely thoughtless. They have failed to distinguish between the conventual vocation and the highly skilled, efficient professional ideal; they need enlightenment, and for this they must have a clear and concise statement presented for their careful consideration. A nation which enjoys an international reputation as to its ability to settle in a sane and satisfactory manner industrial disputes can scarcely fail to grant well-earned concessions to a section of its people who, during past years, have given so freely and asked so little in return; but until the account is presented the debt can never be discharged.

It is these facts the nursing services are apt to forget. Their leaders are primarily responsible for the present lethargic attitude of public opinion, since they have never stated their case, but have avoided discussion and omitted debate from their programme. If they are to continue their evolution they must change their tactics. They must ask for full and free inquiry. The spirit of unrest is leavening within them; their future is in their own hands.

Co-ordination is the crying need of the moment and the ability to subordinate detail to the general scheme, so that a policy may be evolved that will benefit not one but every branch of the nursing services, and by its moderation and its justice stamp itself upon the public imagination. The nursing services are irretrievably bound up with the national life; they are interdependent; their interests are identical; the one cannot exist without the other. It is for members of the nursing services to drive this knowledge home and make the public understand. When they have accomplished this miracle they will have nothing to fear.

